# Novel Markers in Pediatric Acute Lymphoid Leukemia: The Role of ADAM6 in B Cell Leukemia

**DOI:** 10.3389/fcell.2021.706129

**Published:** 2021-06-25

**Authors:** Laila Alsuwaidi, Mahmood Hachim, Abiola Senok

**Affiliations:** ^1^College of Medicine, Mohammed Bin Rashid University of Medicine and Health Sciences, Dubai, United Arab Emirates; ^2^Center for Genomic Discovery, Mohammed Bin Rashid University of Medicine and Health Sciences, Dubai, United Arab Emirates

**Keywords:** ADAM6, acute lymphoid leukemia, bioinformactics analysis, biomarkers, pediatric leukemia

## Abstract

**Background:**

The extensive genetic heterogeneity found in the B cell precursor acute lymphoblastic leukemia (BCP-ALL) subtype of childhood ALL represents a potential repository of biomarkers. To explore this potential, we have carried out *in silico* analysis of publicly available ALL datasets to identify genetic biomarkers for childhood BCP-ALL, which could be used either individually or in combination as markers for early detection, risk stratification, and prognosis.

**Methods:**

To explore novel genes that show promising clinical and molecular signatures, we examined the cBioPortal online tool for publicly available datasets on lymphoid cancers. Three studies on lymphoblastic and lymphoid leukemia with 1706 patients and 2144 samples of which were identified. Only B-Lymphoblastic Leukemia/Lymphoma samples (*n* = 1978) were selected for further analysis. Chromosomal changes were assessed to determine novel genomic loci to analyze clinical and molecular profiles for the leukemia of lymphoid origin using cBioPortal tool.

**Results:**

ADAM6 gene homozygous deletions (HOM:DEL) were present in 59.60% of the profiled patients and were associated with poor ten years of overall patients’ survival. Moreover, patients with ADAM6 HOM:DEL showed a distinguished clinical and molecular profile with higher Central Nervous System (CNS) sites of relapse. In addition, ADAM6 HOM:DEL was significantly associated with unique microRNAs gene expression patterns.

**Conclusion:**

ADAM6 has the potential to be a novel biomarker for the development and progress of BCP- ALL.

## Background

Acute lymphoblastic leukemia (ALL) is a clonal expansion of abnormal lymphoid progenitors of B cell or T cell origin, eventually invading the bone marrow and peripheral blood (Pastorczak et al., 2021). ALL is the most common malignancy in the pediatric age group accounting for 26% of childhood and adolescent cancers (Ward et al., 2014). Although ALL develops in children and adults, the peak incidence is in those aged 1–4 years (Malard and Mohty, 2020). Some cases of pediatric acute leukemias that can be diagnosed in children under one year of age are characterized by unique and aggressive biology (Ibrahimova et al., 2021). On the other hand, 60% of ALL are diagnosed before the age of 20 years (Giddings et al., 2016; Howlader et al., 2021). It represents a significant health concern globally as a major cause of childhood cancer-related mortality affecting children and young adults in their prime age (Starý and Hrušák, 2016; Zapata-Tarrés et al., 2021). From 1975-2012, ALL incidence increased by 0.8% per year in the United States to reach 15.7 cases for every 10^6^ persons, with approximately 5,970 new cases and 1,440 deaths in 2017 (Siegel et al., 2016).

The B cell precursor ALL (BCP-ALL) is a subtype of childhood ALL with a suggested multi-factorial etiology of a mixed inherited and infectious exposure (Greaves, 2018). Notably, BCP-ALL includes many genetic subtypes characterized by significant chromosomal alterations that result in the upregulation of genes by juxtaposition or dysregulation of proteins through the formation of chimeric genes (Malard and Mohty, 2020). These genes include hematopoietic transcription factors, epigenetic modifiers, cytokine receptors, and tyrosine kinases (Malard and Mohty, 2020). BCP-ALL cases usually carry a chromosome translocation as a primary genetic event, plus acquired secondary genetic alterations commonly affect cellular mechanisms that control B-cell differentiation and proliferation(Zhou et al., 2012).

The extensive genetic heterogeneity found within ALL in general, and BCP-ALL specifically represents a potential repository of biomarkers that could be harnessed for the development of early diagnostic and monitoring tools as well as novel chemotherapeutic targets. This is pertinent as cytogenetics plays a significant role in patients’ diagnosis and risk stratification for treatment in childhood BCP-ALL (Schwab et al., 2013). The application of bioinformatics tools enables rapid screening for novel biomarkers, which can be further investigated for prognostic and predictive use to enhance personalized precision treatment. To facilitate this, we have carried out *in silico* analysis of publicly available ALL datasets to identify genetic biomarkers for childhood BCP-ALL, which could be used either individually or in combination as markers for early detection, risk stratification, and prognosis.

## Materials and Methods

### Explore Publicly Available Patients Clinical and Molecular Databases

To explore the novel or less studied genes that show promising clinical and molecular signatures, we examined the cBioPortal online tool for publicly available datasets (Cerami et al., 2012). We searched for lymphoid cancers, and selected the three studies with lymphoblastic and lymphoid leukemia [Acute Lymphoblastic Leukemia (St Jude, Nat Genet 2015), Acute Lymphoblastic Leukemia (St Jude, Nat Genet 2016), and Pediatric Acute Lymphoid Leukemia - Phase II (TARGET, 2018)]. These are based in whole or part based upon data generated by the Therapeutically Applicable Research to Generate Effective Treatments^1^ initiative, phs000218. The data used for this analysis are available at https://portal.gdc.cancer.gov/projects.

**FIGURE 1:**
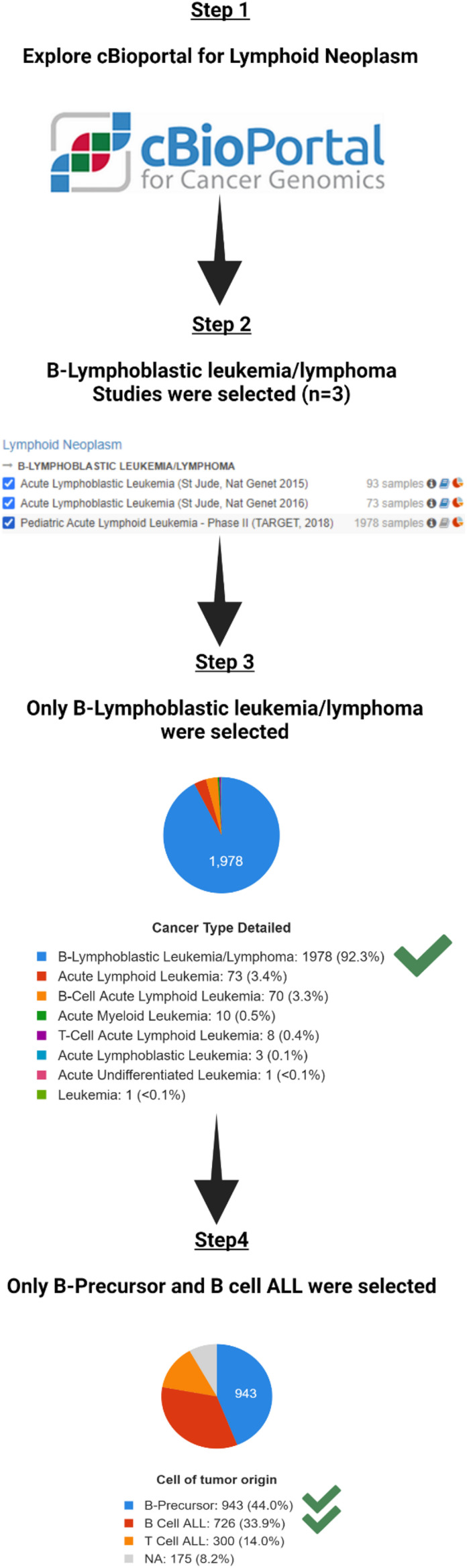
The flowchart for the studies and samples selected for the analysis.

In total, the three studies included 1706 total patients with 2144 samples. B-Lymphoblastic Leukemia/Lymphoma was the largest in the three studies with 1978 samples (Table 1A). To decrease heterogeneity of sample sources, only these 1978 B-Lymphoblastic Leukemia/Lymphoma samples were selected for further analysis. Pediatric Acute Lymphoid Leukemia - Phase II (TARGET, 2018) contains those 1978 samples, so we restricted the further analysis according to the cell of origin in the study, so we excluded those from T cell ALL (*n* = 300) and kept only B-Precursor and B Cell ALL (Table 1B). The flowchart for the studies and samples selected for the analysis is shown in Figure 1.

**TABLE 1A:** Cancer Type Detailed and numbers for lymphoid cancers and specifically the studies with lymphoblastic and Lymphoid leukemia [Acute Lymphoblastic Leukemia (St Jude, Nat Genet 2015), Acute Lymphoblastic Leukemia (St Jude, Nat Genet 2016), and Pediatric Acute Lymphoid Leukemia - Phase II (TARGET, 2018)] used in our study.

Cancer type detailed	Number	Percentage
B-Lymphoblastic leukemia/lymphoma	1978	92.26%
B-Cell acute lymphoid leukemia	70	3.26%
T-Cell acute lymphoid leukemia	8	0.37%
Acute lymphoblastic leukemia	3	0.14%
Leukemia	1	0.05%
Acute undifferentiated leukemia	1	0.05%
Acute myeloid leukemia	10	0.47%
Acute lymphoid leukemia	73	3.40%
Total samples	2144	100.00%
Total patients	1706	

**TABLE 1B:** Pediatric Acute Lymphoid Leukemia - Phase II (TARGET, 2018)] used in our study classified according to their cell of origin.

Category	Number of samples	Percentage of samples
B-precursor	943	47.7%
B cell all	726	36.7%
T cell all	300	15.2%
NA	9	0.5%

### Molecular Differences Between the Identified Groups

For downstream work, we used the cBioPortal tool to analyze the clinical and molecular profiles of patients. Chromosomal changes in the selected samples were assessed to determine if any novel genomic loci for the leukemia of lymphoid origin were identifiable. The subsequent analysis focused on distinguishing the profiles of patients who had the predominant novel genomic loci compared to those with intact or wild counterparts.

### miRNA Targets

To determine the common targets of the identified miRNA, we used miRDB online database for miRNA target prediction and functional annotations^2^. The identified differentially expressed miRNA targets between the groups were intersected with the target of the rest of miRNA, and common targets among at least 4 out of the six miRNAs were selected. TargetScan^3^ was used to predict biological targets of miRNAs by searching for the presence of conserved 8mer, 7mer, and 6mer sites that match the seed region of each miRNA(Agarwal et al., 2015).

## Results

### Identification of the Novel Genomic Loci Significant in Leukemia of Lymphoid Origin

Interestingly we identified nine genes (ADAM6, LINC00226, FAM30A, LINC00221, CDKN2A, CDKN2B, CDKN2B-AS1, MTAP, and PRSS1) which had homozygous deletions present in more than 25% of profiled cases (Table 2). Additionally, most of these genes were found in two common loci where (ADAM6, LINC00226, FAM30A, and LINC00221 in 14q32.33 cytoband) and (CDKN2A, CDKN2B, CDKN2B-AS1, and MTAP in 9p21.3 cytoband). Of these nine genes, only four are known Cancer Genes in OncoKB (CDKN2A, CDKN2B, MTAP, and PRSS1), as shown in Table 2.

**TABLE 2:** Distribution of genes with the highest percentage of chromosomal changes in the profiled samples.

Gene	Cytoband	CNA	Samples with the given CNA	Profiled samples	Percentage	Is cancer gene (source: OncoKB)
ADAM6	*14q32.33*	HOMDEL	455	764	59.60%	No
LINC00226	*14q32.33*	HOMDEL	355	764	46.50%	No
FAM30A	*14q32.33*	HOMDEL	340	764	44.50%	No
LINC00221	*14q32.33*	HOMDEL	307	764	40.20%	No
CDKN2A	9p21.3	HOMDEL	276	764	36.10%	***Yes***
CDKN2B	9p21.3	HOMDEL	257	764	33.60%	***Yes***
CDKN2B-AS1	9p21.3	HOMDEL	236	764	30.90%	No
MTAP	9p21.3	HOMDEL	203	764	26.60%	***Yes***
PRSS1	7q34	HOMDEL	193	764	25.30%	***Yes***

*CNA, copy number aberration.*

**FIGURE 2:**
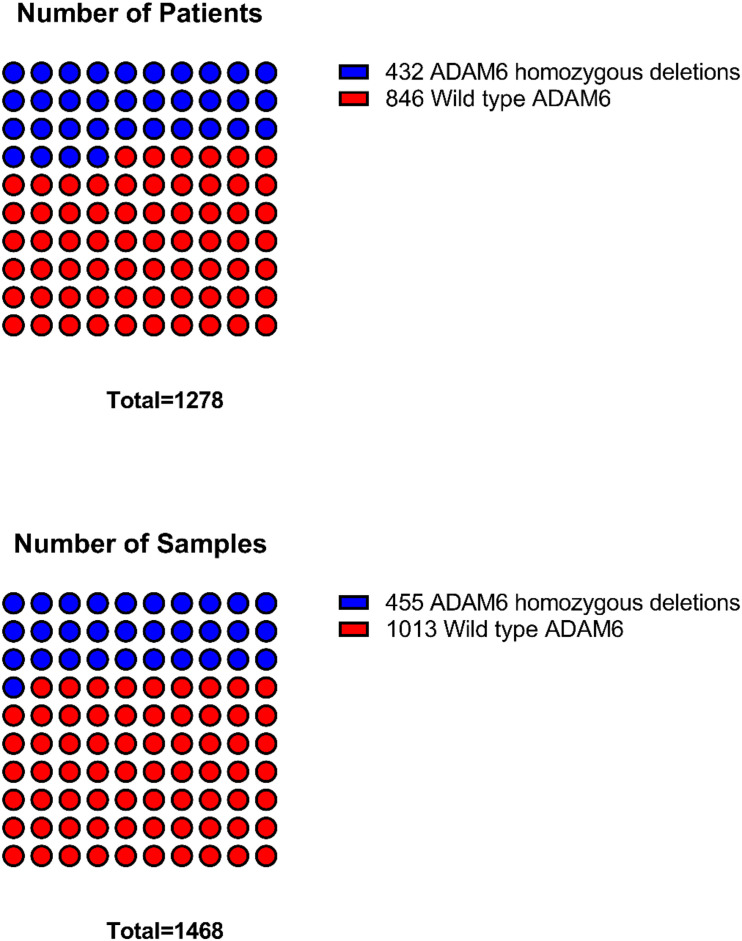
Distribution of samples and patients with ADAM6 homozygous deletions compared to the rest with wild-type ADAM6.

The frequency of ADAM6 homozygous deletion (ADAM6 HOM: DEL) was the highest among the nine genes (455 out of 764; 59.60%); therefore, further analysis was carried out to examine the difference between patients with this deletion (*n* = 455 samples from 432 patients) and the patients without the deletion (*n* = 846 samples from 1013 patients) as shown in Figure 2.

### Patients With ADAM6 HOM:DEL Showed a Distinct Clinical Profile

**FIGURE 3:**
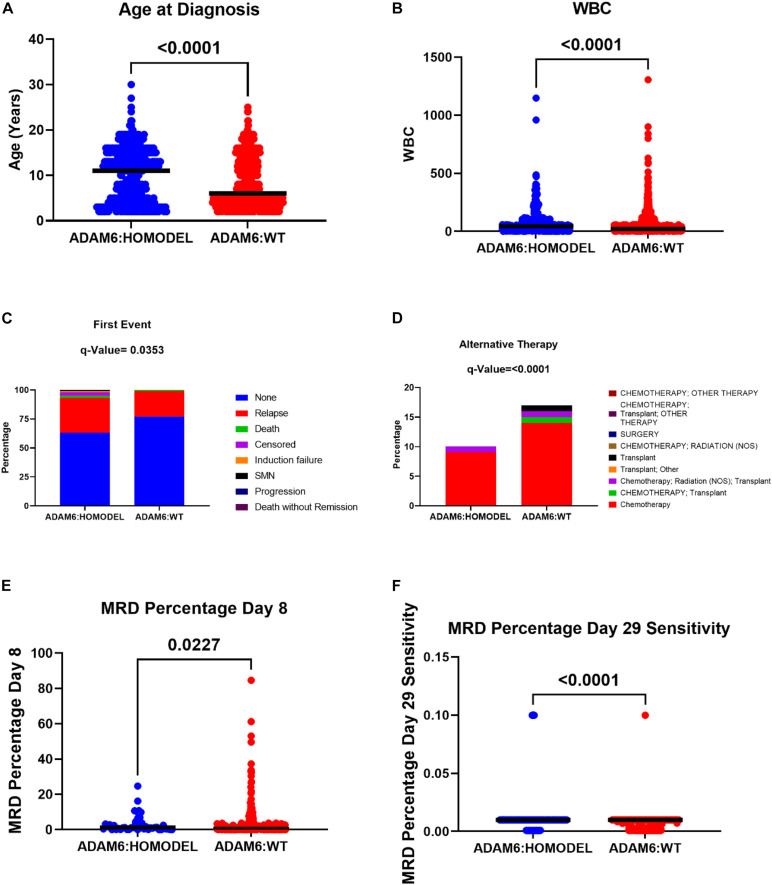
The difference in the clinical attributes between ADAM6:HOMDEL versus WT:ADAM6 patients.

To determine if there were clinical differences between patients with the ADAM6 HOM: DEL and those with intact ADAM6 (wild-type ADAM6 [WT:ADAM6]), the cBioPortal clinical comparison tools was used to compare the two groups. Compared to patients with WT:ADAM6, those with ADAM6 HOM:DEL presented at a later age, higher total WBC count, needed chemotherapy, radiation, and transplant as an alternative therapy, more relapse rate as the first event with higher minimal residual disease (MRD) at day 8 and 29 as shown in Table 3 and Figure 3.

**TABLE 3:** Comparison of clinical attributes of ADAM6 homozygous deletion (ADAM6:HOMDEL) versus ADAM6 wild-type (WT:ADAM6) patients.

Clinical attribute	Attribute type	Statistical test	*p*-Value	*q*-Value
Diagnosis age	Patient	Wilcoxon test	5.85E−07	2.39E−06
Diagnosis age (days)	Patient	Wilcoxon test	1.06E−06	3.99E−06
WBC	Patient	Wilcoxon test	7.42E−05	2.57E−04
Alternative therapy given	Patient	Chi-squared test	9.64E−04	3.10E−03
CNS status	Patient	Chi-squared test	5.54E−08	2.49E−07
First event	Patient	Chi-squared test	0.0157	0.0353
MRD Percentage day 8	Patient	Wilcoxon test	0.0231	0.0472
MRD Percentage day 29 sensitivity	Patient	Wilcoxon test	5.94E−10	5.35E−09

**FIGURE 4:**
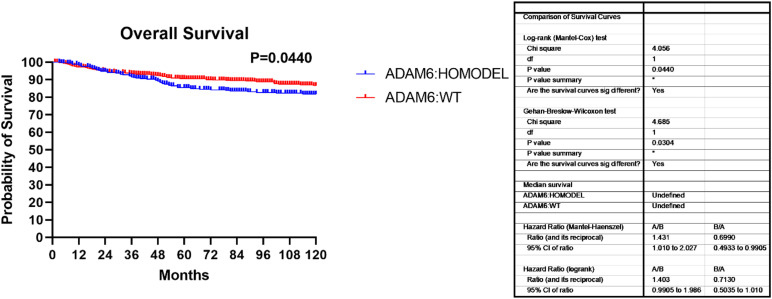
Overall survival showing the ADAM6:HOMDEL versus WT:ADAM6.

Additionally, we found that patients with the ADAM6 HOM:DEL showed poor ten-year survival compared to those with intact ADAM6 (Figure 4).

### ADAM6 HOM:DEL Patients Showed Distinct Molecular Profile

**FIGURE 5:**
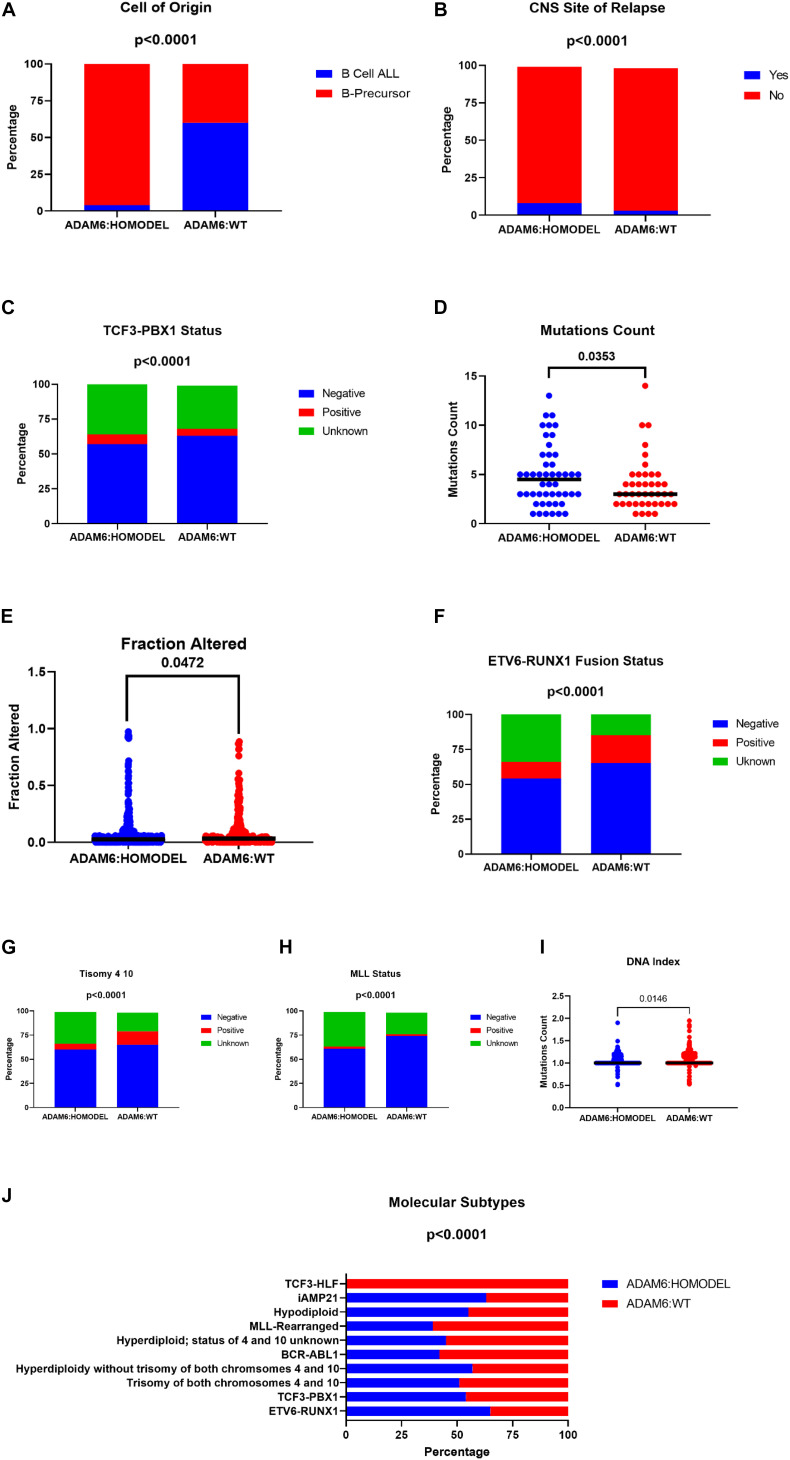
Comparison of Molecular attributes of ADAM6 homozygous deletion (ADAM6:HOMDEL) versus ADAM6 wild-type (WT:ADAM6) patients.

Samples from patients with ADAM6 HOM:DEL had B precursor predominantly as a cell of origin with specific molecular subtypes like higher frequencies of TCF3-PBX1, ETV6-RUNX1 Fusion, and less Trisomy 4_10 status. Furthermore, although ADAM6 HOM:DEL was associated with higher mutation counts and a less altered fraction of the genome, as shown in Table 4 and Figure 5.

**TABLE 4:** Comparison of Molecular attributes of ADAM6 homozygous deletion (ADAM6:HOMDEL) versus ADAM6 wild-type (WT:ADAM6) patients.

Clinical attribute	Attribute type	Statistical test	*p*-Value	*q*-Value
Cell of tumor origin	Sample	Chi-squared test	0	0
TCF3-PBX1 status	Sample	Chi-squared test	8.62E-03	0.0215
Mutation count	Sample	Wilcoxon test	0.0151	0.0353
Fraction genome altered	Sample	Wilcoxon test	0.0221	0.0472
ETV6-RUNX1 fusion status	Sample	Chi-squared test	1.67E−15	1.87E−14
Molecular subtype	Sample	Chi-squared test	3.92E−08	1.96E−07
Trisomy 4_10	Sample	Chi-squared test	6.52E−09	4.19E−08
DNA index	Sample	Chi-squared test	2.05E−03	6.16E−03
MLL status	Sample	Chi-squared test	1.33E−08	7.49E−08

### ADAM6 HOM:DEL Patients Showed Specific Deletion of Other Key Genes

In patients with ADAM6 HOM:DEL, we identified other genes which were also deleted. The top genes with the highest deletion were (FAM30A, LINC00226, LINC00221, VPREB1, PRSS1, PRSS2, BCL2L14, LRP6, and ETV6). Of note, 3 of these genes (FAM30A, LINC00226, and LINC00221) are present in the same cytoband, while the other three genes (BCL2L14, LRP6, and ETV6) are located in the 12p13.2 cytoband. Two genes (PRSS1and PRSS2) are present on 12p13.2, while VPREB1 was the only gene on 22q11.22 as listed in the Table 5.

**TABLE 5:** Most frequently deleted genes in ADAM6 homozygous deletion (ADAM6:HOMDEL) versus ADAM6 wild-type (WT:ADAM6) patients.

Gene	Cytoband	(A) ADAM6:HOMDEL	(B) WT:ADAM6	Log Ratio	*p*-Value	*q*-Value
ADAM6	14q32.33	432 (100.00%)	0 (0.00%)	> 10	7.45E−194	1.24E−189
FAM30A	14q32.33	309 (71.53%)	19 (7.60%)	3.23	2.06E−65	1.72E−61
LINC00226	14q32.33	301 (69.68%)	36 (14.40%)	2.27	4.06E−47	2. 26E−43
LINC00221	14q32.33	249 (57.64%)	48 (19.20%)	1.59	1.29E−23	5.36E−20
VPREB1	22q11.22	128 (29.63%)	20 (8.00%)	1.89	2.90E−12	9.68E−09
PRSS1	7q34	151 (34.95%)	36 (14.40%)	1.28	1.87E−09	5.21E−06
PRSS2	7q34	104 (24.07%)	19 (7.60%)	1.66	1.32E−08	3.14E−05
BCL2L14	12p13.2	57 (13.19%)	5 (2.00%)	2.72	8.57E−08	1.59E−04
LRP6	12p13.2	57 (13.19%)	5 (2.00%)	2.72	8.57E−08	1.59E−04
ETV6	12p13.2	70 (16.20%)	9 (3.60%)	2.17	1.02E−07	1.70E−04

*q-Value < 0.05 significant, ADAM6:HOMDEL, ADAM6 homozygous deletion; WT:ADAM6, ADAM6 wild-type.

### ADAM6 HOM:DEL Is Associated With Unique mRNA and microRNA Genes Expression

**FIGURE 6:**
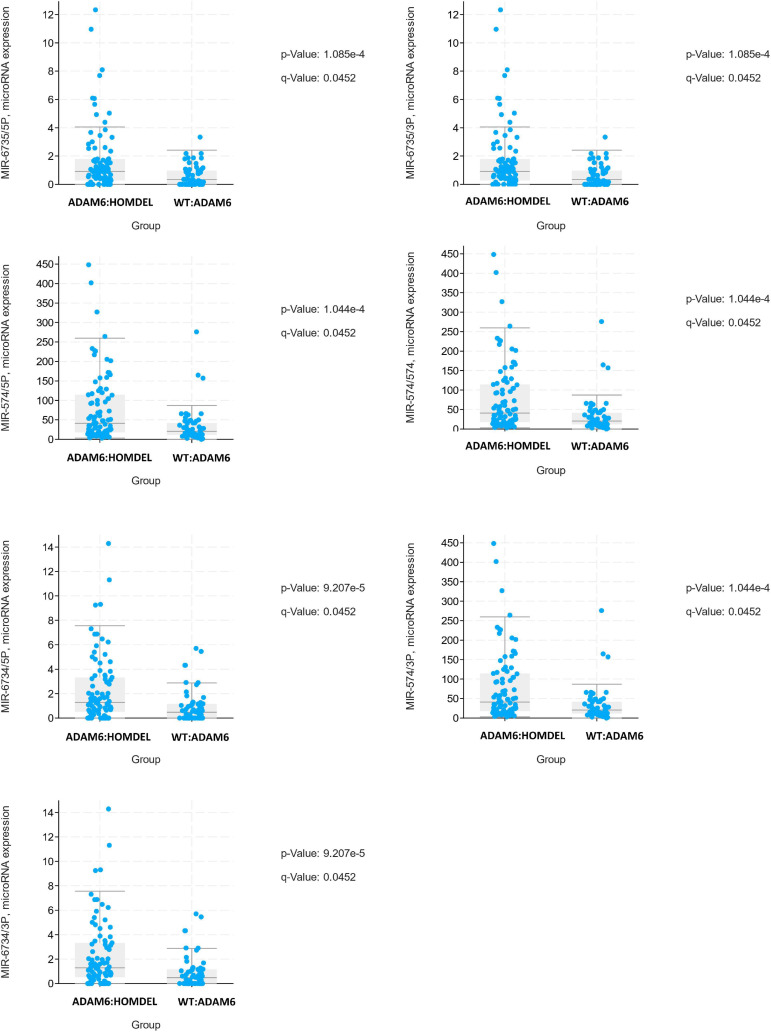
The top statistically different miRNA between ADAM6:HOMDEL versus WT:ADAM6 patients.

To examine if there are specific mRNA changes between patients with ADAM6 HOM:DEL and those without ADAM6 deletion, the mRNA expression (microarray) comparison tool of cBioPortal was used. Our findings showed specific significant differential expression of 1080 genes between the two groups. To examine if there are specific microRNA changes between patients with ADAM6 HOM:DEL and those without ADAM6 deletion, the miRNA comparison tool of cBioPortal was used. Our findings showed specific significant differential expression of 3 pairs of miRNA targets (MIR-574/3P MIR-574/574, MIR-574/5P, MIR-6734/3P, MIR-6734/5P, MIR-6735/3P, and MIR-6735/5P) as shown in Table 6 and Figure 6.

**TABLE 6:** Specific microRNA changes between the ADAM6 HOM:DEL group and the rest of the patients.

Gene	Mean Log2 expression ADAM6:HOMDEL	The standard deviation of lo2 expression ADAM6:HOMDEL	Mean Log2 expression WT:ADAM6	The standard deviation of lo2 expression WT:ADAM6	Log Ratio	*p*-Value	*q*-Value	Higher expression in
MIR-6735/3P	1.7	2.35	0.61	0.74	1.09	1.09E−04	0.0452	ADAM6:HOMDEL
MIR-6735/5P	1.7	2.35	0.61	0.74	1.09	1.09E−04	0.0452	ADAM6:HOMDEL
MIR-6734/3P	2.32	2.76	0.92	1.34	1.4	9.21E−05	0.0452	ADAM6:HOMDEL
MIR-6734/5P	2.32	2.76	0.92	1.34	1.4	9.21E−05	0.0452	ADAM6:HOMDEL
MIR-574/3P	78.95	89.45	33.81	44.55	> 10	1.04E−04	0.0452	ADAM6:HOMDEL
MIR-574/574	78.95	89.45	33.81	44.55	> 10	1.04E−04	0.0452	ADAM6:HOMDEL
MIR-574/5P	78.95	89.45	33.81	44.55	> 10	1.04E−04	0.0452	ADAM6:HOMDEL

*q-Value < 0.05 significant, ADAM6:HOMDEL, ADAM6 homozygous deletion; WT:ADAM6, ADAM6 wild-type.

MicroRNAs (miRNAs) are small non-coding RNAs that act as master regulators of the expression of their gene targets, so predicting miRNA targets is a vital step in the characterization of miRNA functions (Chen and Wang, 2019). We surmised that if the miRNA target the same genes, we might identify a novel mechanism that controls the Differentially expressed genes (DEGs) between the two groups. Therefore, to determine the common targets of the identified miRNA, we used miRDB online database for miRNA target prediction and functional annotations^2^ for analysis. The target of each miRNA was intersected with the target of the rest miRNA and common targets among at least 4 out of the six miRNAs were selected (Table 7).

**TABLE 7:** Shared targets by the specific microRNA changed between the ADAM6 HOM:DEL group and the rest of the patients.

[hsa-miR-6735-3p] and [hsa-miR-6735-5] and [hsa-miR-6734-3] and [hsa-miR-6734-5p]	CCDC85C, ACVR2B, NOVA2, TBC1D16, FKTN, STIM1, HLA-DQA1, C6orf106
[hsa-miR-6735-3p] and [hsa-miR-6735-5] and [hsa-miR-574-5p] and [hsa-miR-6734-3]	KIAA0513, TMEM106A, DCAF8, SEMA7A
[hsa-miR-6735-3p] and [hsa-miR-6735-5] and [hsa-miR-574-5p] and [hsa-miR-6734-5p]	NOS1, NTRK2, FOXN3, MASP1
[hsa-miR-6735-3p] and [hsa-miR-6735-5] and [hsa-miR-574-5p] and [hsa-miR-6734-3] and [hsa-miR-6734-5p]	MEF2C

Our findings show that myocyte-enhancer factor 2 C (MEF2C) was common between 5 miRNA targets. In order to identify the putative binding sequence of the target gene (MEF2C) and binding scores, we used targetscan online tool^3^ to search for MEF2C-miRNA interactions. The identifier MEF2C corresponds to 2 transcripts. One is the representative (most prevalent) transcript for MEF2C (ENST00000340208.5) and The second is the less prevalent transcript for MEF2C (ENST00000506554.1). The MEF2C-miRNA interactions, binding sites, and scores are shown in Table 8.

**TABLE 8:** The MEF2C-miRNA interactions, binding sites and scores as per Targetscan tools.

TargetScan_7.2 ENST00000340208.5	Position in the UTR	seed match	context + + score	context + + score percentile	weighted context + + score	conserved branch length
hsa-miR-6734-3p	65–71	7mer-m8	−0.12	79	−0.12	0
hsa-miR-574-5p	2370–2377	8mer	−0.27	90	−0.02	0
hsa-miR-6734-3p	2814–2820	7mer-m8	−0.2	89	−0.02	0
hsa-miR-6735-5p	3427–3433	7mer-m8	−0.1	69	−0.01	0.322
hsa-miR-6734-5p	3595–3601	7mer-1A	−0.11	67	−0.01	0.159

**TargetScan_7.2 ENST00000506554.1**	**Position in the UTR**	**seed match**	**context + + score**	**context + + score percentile**	**weighted context + + score**	**conserved branch length**

hsa-miR-6734-3p	214–220	7mer-m8	−0.11	76	−0.11	0
hsa-miR-574-5p	2519–2526	8mer	−0.28	91	0	0
hsa-miR-6735-5p	3576–3582	7mer-m8	−0.11	71	0	0.322
hsa-miR-6734-5p	3744-3750	7mer-1A	−0.11	68	0	0.159

**FIGURE 7:**
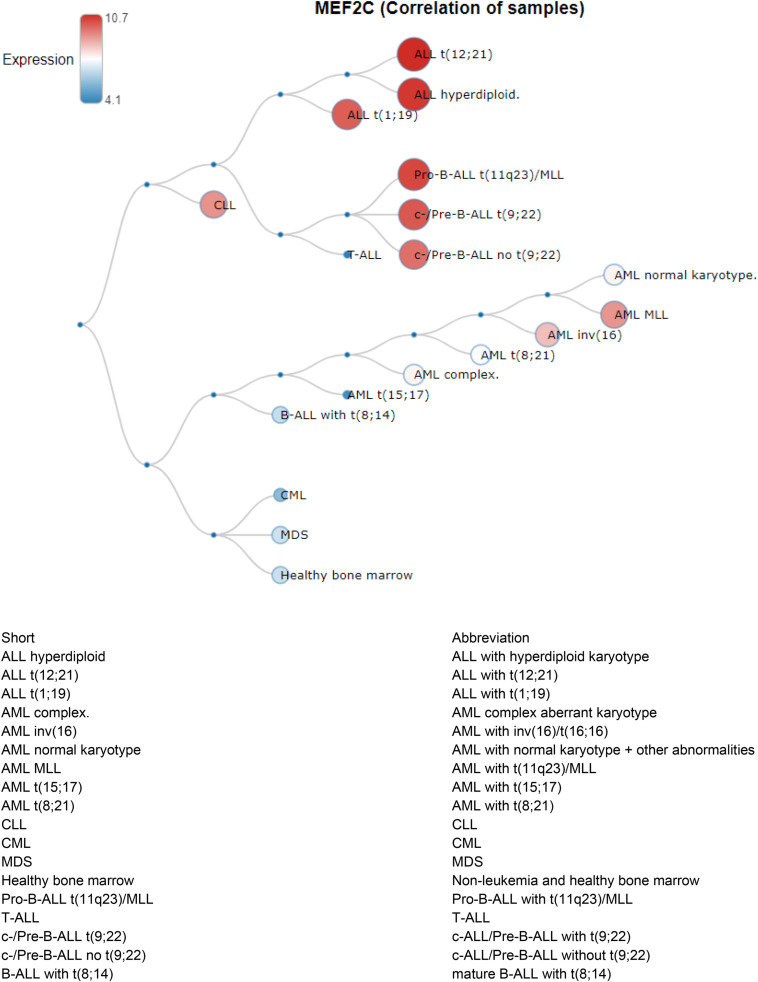
MEF2C (Correlation of samples) in Human AML, ALL and preleukemic stages cells are from GSE13159.

In order to understand the correlation between MEF2C and different leukemias, we explored BloodSpot^4^ “a database of gene expression profiles and transcriptional programs for healthy and malignant hematopoiesis”(Bagger et al., 2015) looking for MEF2C expression. MEF2C expression showed its specificity to ALL, as shown in Figure 7.

### PRSS1, ACOT11, NTRK2, NOVA2, and SEMA7A Might Be the Novel Players in ADAM6 HOM:DEL ALL

**FIGURE 8:**
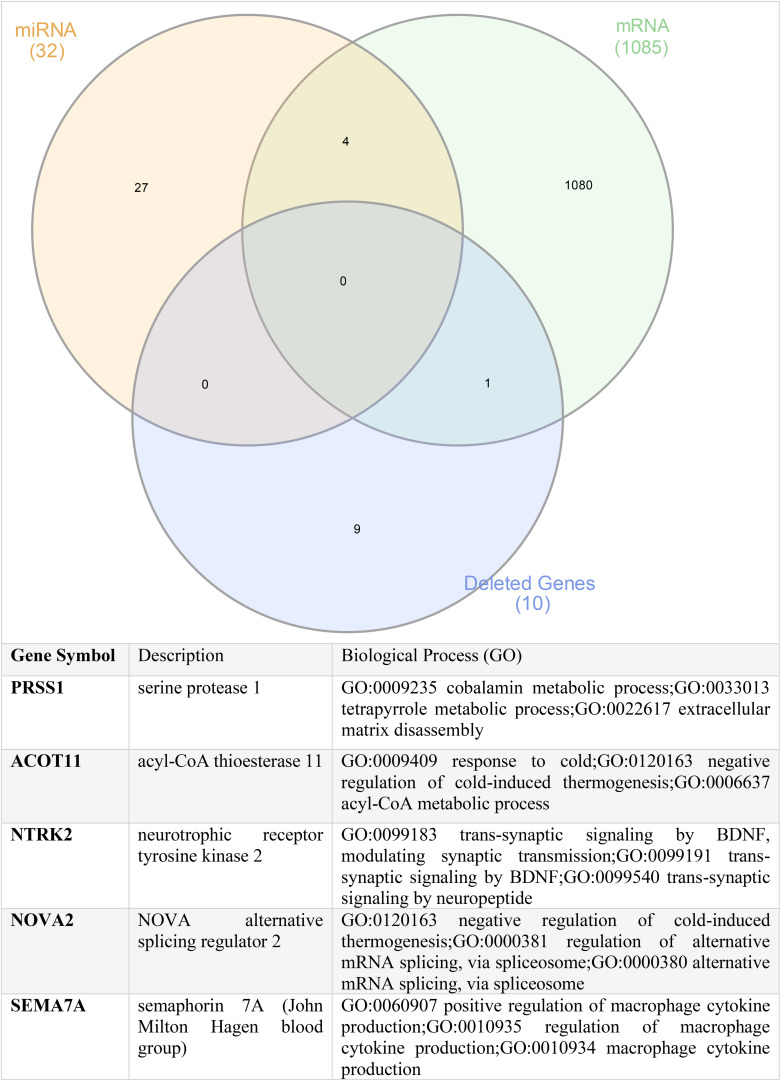
Shared genes between the commonly deleted genes, upregulated mRNA genes, and common targets of the altered miRNA.

Finally, to determine if common players link genomics, transcriptomics, and miRNA together, we intersected the commonly deleted genes, the DEGs, and the shared miRNA targets. Our findings identified the common genes as *PRSS1*, *ACOT11*, *NTRK2*, *NOVA2*, and *SEMA7A*, suggesting that they might be the novel players in ADAM6 HOM:DEL ALL as shown in Figure 8.

## Discussion

Our findings demonstrate that patients with ADAM6 HOM:DEL had a distinct clinical profile compared to those with intact ADAM6. These findings suggest a possible role for ADAM6 in disease pathogenesis, progression, and patient survival. The finding of significant differences in the 10-year survival rate between patients with ADAM6 HOM:DEL *versus* those with intact ADAM6 is of interest as this could potentially be used as a novel biomarker for monitoring the development and progression of BCP-ALL. ADAM6 is a member of a disintegrin and metalloproteinases (ADAMs) gene family of proteins which display a common domain organization featuring a pro-domain, a metalloprotease, a disintegrin, a cysteine-rich, an epidermal growth factor-like, and a transmembrane domain, as well as a C-terminal cytoplasmic tail (Wolfsberg et al., 1995; Brocker et al., 2009). ADAM members are multifunctional proteins involved in the proteolytic processing of other transmembrane proteins, cell adhesion, and cell signaling events (Weber and Saftig, 2012). Several reports have shown that members of the ADAM family are overexpressed in human cancers such as ADAM8 in human renal cell carcinomas, ADAM15 in lung carcinoma, and ADAM17 in breast cancers (Mochizuki and Okada, 2007). Differences in the active site sequence of the metalloproteinase domain indicate that 60% of ADAM members are pseudogenes and non−proteolytic molecules; as a result, several members of the ADAM family, including ADAM6, a pseudogene located in chromosome 14 (14q32.33), have hitherto not been well studied or reported on in the literature (Mochizuki and Okada, 2007). Indeed, the function of ADAM6 in disease or normal physiological scenarios is yet to be fully elucidated and our findings are an essential contribution to the paucity of data on ADAM6.

Interestingly, our study noted specific microRNA changes in patients with ADAM6 HOM:DEL where the expression of *MIR-574/3P* gene was observed in most ADAM6 HOM:DEL suggesting an association between ADAM6 deletion and the overexpression of the gene. Since *MIR-574/3P* gene is known to suppress proliferation and induces apoptosis of chronic myeloid leukemia (CML) cells via targeting IL6/JAK/STAT3 pathway (Yang et al., 2018), the findings of this study provide novel insights into the association of ADAM6 with miR-574-3p signaling pathway in leukemia. Furthermore, the findings demonstrate that the deletion of ADAM6 is associated with unique microRNA genes expression with significant differential expression of 3 pairs of miRNA targets. The *MEF2C* gene was the one most linked showing an association with 5 of these *miRNA* targets. *MEF2C* is necessary for the proper development of megakaryocytes and platelets and bone marrow B-lymphopoiesis. Moreover, *MEF2C* is required for B-cell survival and proliferation in response to B cell receptor (BCR) stimulation, efficient IgG1 antibody responses to T-cell-dependent antigens, and normal germinal center induction B-cells. This gene is a selectively expressed transcription factor that, if ectopically expressed due to chromosomal rearrangements, can lead to mixed-lineage leukemia-rearranged acute myeloid leukemia and immature T-cell acute lymphoblastic leukemia (Canté-Barrett et al., 2014). Phosphorylation of *MEF2C* has been reported in the majority of primary chemotherapy-resistant AML (Brown et al., 2018). Its high expression is linked with a subset of AML patients with adverse-risk disease features and poor outcomes, with confirmation that high MEF2C mRNA expression leads to overexpression of MEF2C protein (Laszlo et al., 2015). These findings provided the rationale for the therapeutic targeting of MEF2C transcriptional activation in AML. The finding of *MEF2C* as a common link among the miRNA targets in *ADAM6 HOM:DEL* patients suggests the potential role for this gene in disease progression, which warrants further investigation of its use as a biomarker.

Our findings also demonstrate that the *PRSS1*, *ACOT11*, *NTRK2*, *NOVA2*, and *SEMA7A* genes were common denominators linking the unique genomic, transcriptomic, and miRNA profiles identified in the ADAM6 HOM:DEL ALL patients. This is pertinent as *PRSS1*, *ACOT11*, *NTRK2* have been shown individually to play a role in other hematopoietic and solid organ malignancies but have hitherto not been previously reported in the context of BCP-ALL. Trypsin-encoding *PRSS1-PRSS2* variations influence the risk of asparaginase-associated pancreatitis in children with acute lymphoblastic leukemia (Wolthers et al., 2019). Recent genome-wide association studies have found different candidate single-nucleotide polymorphisms associated with pancreatitis in patients with ALL (Rank et al., 2019). Acyl-CoA Thioesterase 11 (ACOT11) is a protein-coding gene, and its high expression in patients with lung adenocarcinoma was associated with cell proliferation and poor prognosis (Hung et al., 2017). In AML, high ACOT11 expression was associated with poor overall survival (Luo et al., 2016). In clear (ccRCC), ACOT11 has been identified as a diagnostic marker wherein mRNA level of *ACOT11* was decreased compared to those in normal kidneys (Luo et al., 2016). Neurotrophic receptor tyrosine kinase 2 (NTRK2) is a kinase target for differentially expressed glutathione peroxidases (GPX-8) in AML (Wei et al., 2020). NTRK2 has been implicated in several types of cancers, including neuroblastoma, medulloblastoma, Wilm’s tumor, and adenocarcinomas of the lung, prostate, and pancreas as well as multiple myeloma (Yuzugullu et al., 2016). In hematopoietic cells, B and T lymphocytes and monocytes have been shown to produce NTRK2 ligand (Kerschensteiner et al., 1999). NTRK2 and its ligand, brain-derived neurotrophic factors (BDNF), are co-expressed in acute leukemia blasts and negatively correlate with leukemia patients’ survival. Studies have shown that mice with bone marrow transduced with both NTRK2 and BDNF developed AML and T-ALL (Uren and Turnley, 2014). NTRK2 overexpression is enriched in a subset of PTEN-deficient T-ALL (Yuzugullu et al., 2016). Additionally, NTRK2 activation cooperates with PTEN deficiency to promote the proliferation of Ba/F3 cells in the absence of IL3 in T-ALL (Yuzugullu et al., 2016).

Our assessment of the clinical profiles showed that patients with ADAM6 HOM:DEL were more likely to have the CNS as the site of relapse. This is interesting as two genes, namely *NOVA2* and SEMA7A, were among common players linking the unique genomic, transcriptomic, and miRNA profiles in ADAM6 HOM:DEL ALL patients play important roles in CNS development and regulation. Neuro-Oncological Ventral Antigen 2 (*NOVA2*) is one of two NOVA proteins involved in neuronal-specific alternative splicing and is mainly expressed in the cerebral cortex and hippocampus (Yang et al., 1998). *NOVA2* seems to be mainly associated with a splicing regulation of genes involved in axonal guidance and projection during the development of the cortex and genes implicated in the cerebellar function of synapse formation (Saito et al., 2016). It regulates a series of alternative splicing events linked to neurite outgrowth and axonal projections in human neural stem cells (Mattioli et al., 2020). Semaphorin 7A (*SEMA7A*) is a membrane-anchored protein that plays a critical role in neuronal pathfinding and axon guidance in selected areas of the developing nervous system. It is also known to be expressed widely with diverse functions, including immune cell modulation, bone remodeling, and drug resistance (Yazdani and Terman, 2006). SEMA7A is the only GPI-linked protein in the semaphorin family that is prominently expressed in the embryo, in the lymphoid organs, and in the nervous system of adult mice (Sato and Takahashi, 1998), it enhances central and peripheral axon growth and is required for proper axon tract formation during embryonic development (Pasterkamp et al., 2003). Though the precise function of Sema7A in the immune system remains unclear, Sema7A has been shown to stimulate human monocytes (Holmes et al., 2002), and its function as a negative regulator of T-cell responses has also been reported (Czopik et al., 2006).

Overall, our findings provide the first insight to indicate that these genes might work together in the context of ADAM6 deletion. We hypothesize that they might play critical roles in determining the disease pathogenesis and progression in patients with deletion of ADAM6. This is in keeping with the poor patient survival trends demonstrated for ADAM6 HOM:DEL. Therefore, the identification ADAM6 deletion in BCP-ALL can be used as a genetic biomarker disease progression and prognosis.

## Conclusion

The findings suggest that ADAM6 can be a novel genetic biomarker for risk stratification and prognosis of childhood BCP-ALL. The ADAM6 HOM:DEL is significantly associated with unique microRNA gene expression and is significantly associated with the deletion of essential genes. More research on the underlying signal to explore the molecular pathways and interacting genes is warranted. Comparing ALL patients with and without ADAM6 HOM:DEL in the prospective study can evaluate the predictive value of this promising marker, and making stable lymphoid cancer cells carrying such deletion to compare its biological and signaling derangement might explore such novel molecular changes related to ALL.

## Data Availability Statement

The datasets presented in this study can be found in online repositories. The names of the repository/repositories and accession number(s) can be found in the article/supplementary material.

## Author Contributions

All authors listed have made a substantial, direct and intellectual contribution to the work, and approved it for publication.

## Conflict of Interest

The authors declare that the research was conducted in the absence of any commercial or financial relationships that could be construed as a potential conflict of interest.

## Footnotes

1 https://ocg.cancer.gov/programs/target

2 http://mirdb.org/mirdb/

3 http://www.targetscan.org/vert_72/

4 http://servers.binf.ku.dk/bloodspot/
